# Hypoxia induces the translocation of glucose transporter 1 to the plasma membrane in vascular endothelial cells

**DOI:** 10.1186/s12576-020-00773-y

**Published:** 2020-09-22

**Authors:** Abdullah Al Mamun, Hisaki Hayashi, Aya Yamamura, Md Junayed Nayeem, Motohiko Sato

**Affiliations:** 1 Department of Physiology, Aichi Medical University, 1-1 Yazako-Karimata, Nagakute-City, Aichi, 4801165 Japan; 2 Department of Biochemistry and Molecular Biology, Mawlana Bhashani Science and Technology University, Santosh, Tangail, 1902 Bangladesh

**Keywords:** Glucose transporters, Hypoxia, Endothelial cells, ATP, ATP-sensitive potassium channel

## Abstract

Glucose uptake and adenosine triphosphate (ATP) generation are important for the survival and growth of endothelial cells. An increase of glucose uptake under hypoxia was previously shown to be associated with the increased expression of glucose transporters (GLUTs). However, the regulation of GLUT trafficking to the cell surface has not been examined in detail. Here, we report the characterization of GLUT1 translocation to the plasma membrane during hypoxia in endothelial cells. Human umbilical vein endothelial cells (HUVECs) were exposed to hypoxia (1% O_2_) for 12 h, which significantly induced GLUT1 expression and translocation to the plasma membrane. GLUT1 translocation was associated with a decrease of intracellular ATP by hypoxia. Decreasing ATP levels with antimycin-A and 2-deoxyglucose induced GLUT1 translocation under normoxia. The induction of hypoxia-inducible factor-1α under normoxia did not influence the cell surface expression of GLUT1 or cellular ATP concentration. Interestingly, the translocation of GLUT1 induced by hypoxia was inhibited by the ATP-sensitive potassium (KATP) channel inhibitor glibenclamide, while the mitochondrial KATP channel inhibitor 5-HD did not influence GLUT1 translocation during hypoxia. These observations indicate that a decrease of intracellular ATP triggers GLUT1 translocation to the plasma membrane and is mediated by KATP channels, which would contribute to glucose uptake in HUVECs during hypoxia.

## Introduction

Glucose is the essential source of energy for most cells. Glucose and other carbohydrates are transported from the surrounding fluid into cells by a family of membrane integral proteins called glucose transporters (GLUTs) [1]. To date, 14 GLUTs have been identified in humans, with GLUT1, GLUT3, and GLUT4 being the most commonly expressed in a wide variety of cells [1, 2]. Physiological stimuli induce GLUT expression to facilitate the incorporation of an appropriate quantity of glucose for energy production. For example, hypoxia stimulates glucose transport into cells by increasing the expression of GLUT1 [3, 4]. These processes are regulated to maintain homeostasis, and abnormal expression of GLUTs is often associated with human diseases [2]. The elevated expression of GLUT1 and/or GLUT3 is associated with poor survival in many solid cancers, including gastric cancer [5], breast cancer [6], lung cancer [7], and glioblastoma [8]. In addition, the cell surface localization of GLUTs is an important factor for glucose transport [2]. The insulin-mediated translocation of GLUT4 to the plasma membrane has been well characterized; however, the translocation mechanisms of other GLUTs have not been examined in detail.

Glucose uptake is critical for the growth of endothelial cells and angiogenesis, because energy production in endothelial cells is highly dependent on glycolysis [9, 10]. We previously demonstrated that the concentration of adenosine triphosphate (ATP) is a critical determinant of vascular endothelial growth factor receptor (VEGFR) signaling under hypoxia, which is important for angiogenesis [11]. Thus, under hypoxic conditions, the VEGF-stimulated phosphorylation of VEGFR-2 is significantly inhibited, which is associated with a decrease of intracellular ATP, while the restoration of ATP levels recovers VEGFR-2 signaling. Furthermore, GLUT1 expression is increased by the exposure of endothelial cells to hypoxia [11]. However, the subcellular localization of GLUTs in endothelial cells, particularly under hypoxia, has not been examined in detail. This information is important for our understanding of angiogenic events when confronted with physiological challenges.

In this study, we examined the expression and intracellular localization of GLUT1 under hypoxia, particularly the trafficking of GLUT1 to the cell surface, which is important for glucose uptake by endothelial cells.

## Materials and methods

### Materials

Human umbilical vein endothelial cells (HUVECs) were purchased from Lonza (Basel, Switzerland). A ReverTra Ace RT Kit was bought from TOYOBO (Osaka, Japan). Anti-GLUT1 (#12939) and Alexa Fluor 488 anti-rabbit IgG antibodies were purchased from Cell Signaling Technology (Danvers, MA). Anti-GLUT3 (PA5-72331) was obtained from Thermo Fisher (Waltham, MA). β-actin antibody (AC-74), ATP-sensitive potassium (KATP) channel inhibitor glibenclamide, mitochondrial KATP channel inhibitor 5-HD, ATP inhibitor antimycin-A, 2-deoxyglucose (2-DG), and GLUT1 inhibitor BAY876 were acquired from Sigma-Aldrich (St. Louis, MO). The selectivity of BAY876 for GLUT1 is over 100 times greater than the IC_50_ for GLUT2, GLUT3, and GLUT4 [12]. An ATP Standard and ATP Assay Kit were obtained from TOYO B-Net (Tokyo, Japan). A Glucose Uptake Assay Kit was purchased from Cosmo Bio Co., Ltd. (#MBR-PMG-K01E; Tokyo, Japan). Low-oxygen-containing gas (1% O_2_, 5% CO_2_, and 94% N_2_) was obtained from Daiwa Shokai (Nagoya, Japan).

### Cell culture

HUVECs were cultured in endothelial cell growth medium (EGM-2; Lonza, Basel, Switzerland) on fibronectin-coated culture dishes or plates (Corning, Schiphol, Netherlands). The cells were used between passages 3 and 7 for experiments. HUVECs were cultured in 6-, 12-, or 96-well plates to 70%–80% confluence. The cells were treated with glibenclamide (10 µM) or 5-HD (100 µM) for 1 h and exposed to hypoxia (1% O_2_, 5% CO_2_ at 37 °C) for 12 h or cultured with antimycin-A (10 µM) or 2-DG (1 mM) for 30 min. HUVECs were also treated with CoCl_2_ (100 µM) and cultured for 3 h to mimic hypoxia. Hypoxia was maintained using a hypoxic chamber (AnaeroPack Rectangular Jar; MITSUBISHI GAS CHEMICAL COMPANY, Inc., Tokyo, Japan) with an oxygen monitor (OXY-1; ICHINEN JIKCO Co., Ltd., Tokyo, Japan).

### Real-time PCR

Total RNA was extracted from HUVECs using an RNeasy Mini Kit (Qiagen, Hilden, Germany) according to the manufacturer’s protocol and reverse transcribed to cDNA with the use of a ReverTra Ace RT Kit (TOYOBO). Real-time PCRs were run on a StepOnePlus system (Applied Biosystems, Foster City, CA). The following primers were used for PCR [11, 13]: GLUT1 forward, 5′-CGTCTTCATCATCTTCAC TG-3′; GLUT1 reverse, 5′-CTCCTCGGG TGTCTTATC-3′; GLUT2 forward, 5′-CACACAAGACCTGGAATTGACA-3′; GLUT2 reverse, 5′-CGGTCATCCAGTGGAAGAC-3′; GLUT3 forward, 5′-CGGCTTCCTCATTAC CTTC-3′; GLUT3 reverse, 5′-GGCACGACTTAGACATTGG-3′; GLUT4 forward, 5′-CTGGGCCTCACAGTGCTAC-3′; GLUT4 reverse, 5′-GTCAGGCGCTTCAGACTCTT-3′; GLUT5 forward, 5′-CATCACTGTTGGCATCCTTGTG-3′; GLUT5 reverse, 5′-AGGATCGGCCAGCCATCTAC-3′; Kir6.1 forward, 5′-CAACTGCTGTGTCCAGAT-3′; Kir6.1 reverse, 5′-ATACGAATGGTGATGTTGGA-3′; Kir6.2 forward, 5′-CATAGGCATTAGTGTAGT-3′; Kir6.2 reverse, 5′-TTATAGAAGAGGCAACTG-3′; SUR1 forward, 5′-CAACTGCTGTGTCCAGAT-3′; SUR1 reverse, 5′-ATACGAATGGTGATGTTGGA-3′; SUR2A forward, 5′-AAGCATTCGGTCATTGTAG-3′; SUR2A reverse, 5′-GCCACATAGTAGGTCTGA-3′; SUR2B forward, 5′-TGGAGAGGATGTGGAGAA-3′; SUR2B reverse, 5′-CTGTAAGAATGGTGAATGTGA T-3′; 18S rRNA forward, 5′-GTCTGTGATGCCCTTAGATG-3′; and 18S rRNA reverse, 5′-AGCTTATGACCCGCACTT AC-3′.

### Immunoblot analysis

Total protein lysates were prepared by collecting experimental HUVECs in 1 × sodium dodecyl sulfate (SDS)-polyacrylamide gel electrophoresis (PAGE) sample buffer (65.8 mM Tris–HCl, pH 6.8, 2.1% SDS, 26.3% glycerol, 0.01% bromophenol blue) with protease inhibitor cocktail (Roche, Indianapolis, IN) and 4% 2-mercaptoethanol. The protein lysates were homogenized with a 26 G syringe needle and separated by SDS-PAGE. The separated proteins were transferred onto a PVDF membrane (Millipore, Billerica, MA). The membrane was blocked with Tris-buffered saline containing 5% bovine serum albumin (BSA; Sigma-Aldrich) and incubated with primary antibodies overnight at 4 °C. The membrane was treated with horseradish peroxidase-conjugated secondary antibodies (GE Healthcare, Munich, Germany), and signals were detected using Immunostar LD western blotting detection reagents (Wako Pure Chemical Industries, Osaka, Japan) and a LAS 4000 image analyzer (GE Healthcare). For quantification, band intensity was analyzed with ImageJ software.

### Immunocytochemistry

GLUT1 localization was determined by immunocytochemistry analysis based on a laboratory and antibody recommended protocol [14]. HUVECs were cultured on 13-mm poly-l-lysine-coated coverslips (Matsunami Glass, Osaka, Japan). The cells were washed with ice-cold phosphate-buffered saline (PBS), fixed with 4% paraformaldehyde in PBS for 10 min, and incubated with 1% BSA and 0.3 M glycine in 0.1% PBS-Tween for 1 h to permeabilize the cells and to block nonspecific protein–protein interactions. The cells were incubated with an anti-GLUT1 antibody (#ab652, 1/200 dilution) overnight at 4 °C. The cells were washed with PBS and incubated with Alexa Fluor 488 anti-rabbit IgG (green, 1/500 dilution) for 1 h in the dark. The cells were washed and incubated with DAPI (Life Technologies, Carlsbad, CA) in PBS for 5 min. The cells were washed and mounted on slides with glass coverslips using Fluoro Keeper Antifade Reagent (Nacalai Tesque, Kyoto, Japan). Images were obtained with an LSM710 laser confocal microscope (Carl Zeiss, Jena, Germany).

### Flow cytometric analysis

HUVECs were cultured in 6-well plates with EGM-2 to 70%–80% confluence. A FACS Canto II (BD Biosciences, Franklin Lakes, NJ) or BD LSRFortessa X-20 (BD Biosciences) was used to perform fluorescence-activated cell sorting (FACS). The cells were washed with ice-cold PBS, detached from the plates using Accutase (Innovative Cell Technologies, San Diego, CA), centrifuged, and washed with ice-cold PBS. The cells were fixed with 4% paraformaldehyde for 15 min, washed with PBS, and labeled with a human anti-GLUT1 Alexa Fluor 700-conjugated antibody (FAB1418N, 1:100 dilution; R&D Systems) or a human anti-GLUT1 Alexa Fluor 488-conjugated antibody (FAB1418G, 1:100 dilution; R&D Systems) for 1 h on ice in the dark to detect cell surface GLUT1. Flow cytometric analysis and data acquisition were performed with the Canto II system or BD LSRFortessa X-20 system using FACS Diva software (BD Biosciences). FlowJo software (Tree Star, Ashland, OR) was used for data analysis.

### ATP analysis

To analyze ATP levels in HUVECs, the cells were seeded in fibronectin-coated 96-well plates and cultured with EGM-2 overnight. For the ATP assay, the cells were treated with glibenclamide for 1 h and cultured under normoxia or exposed to hypoxia for 12 h or cultured with CoCl_2_ for 3 h or antimycin-A (10 µM) or 2-DG (1 mM) for 30 min before measuring ATP concentration. Intracellular ATP concentration was measured using a luminescence-based ATP assay kit (TOYO B-Net) according to the manufacturer’s instructions.

### 2-DG uptake assay

Glucose transport into HUVECs was measured using 2-DG with a Glucose Cellular Uptake Measurement Kit (Cosmo Bio Co., Ltd.) according to the manufacturer’s instructions. HUVECs cultured in 12-well plates were washed with warm KRPH buffer (1.2 mM KH_2_PO_4_, 1.2 mM MgSO_4_, 1.3 mM CaCl_2_, 118 mM NaCl, 5 mM KCl, 30 mM HEPES, pH 7.5) containing 2% BSA. The cells were incubated with 1 mM of 2-DG in KRPH buffer for 20 min at 37 °C. Cellular 2-DG uptake was stopped by washing the cells with ice-cold PBS containing 200 µM phloretin (Sigma-Aldrich). Cell lysates were collected in 1 × sample buffer and heated at 80 °C for 15 min. The lysates were centrifuged at 15,000×*g* for 20 min at 4 °C and the supernatants were collected. 2-DG uptake was measured using a fluorometric-based glucose uptake assay kit (Cosmo Bio Co., Ltd.) according to the manufacturer’s instructions.

### Miscellaneous procedures and data analysis

Further details on the miscellaneous procedures are provided elsewhere [15, 16]. Data are expressed as the mean ± standard error of the mean (SEM) from independent experiments. Statistical analysis was performed using an unpaired *t* test, Mann–Whitney test, or one-way analysis of variance (ANOVA) with post hoc analysis. Significant differences between the control and test groups were evaluated with P-values less than 0.05 and 0.01. All statistical analyses were performed with IBM SPSS software (Armonk, NY) [17].

## Results

### Hypoxia induces glucose uptake in HUVECs

**Fig. 1:**
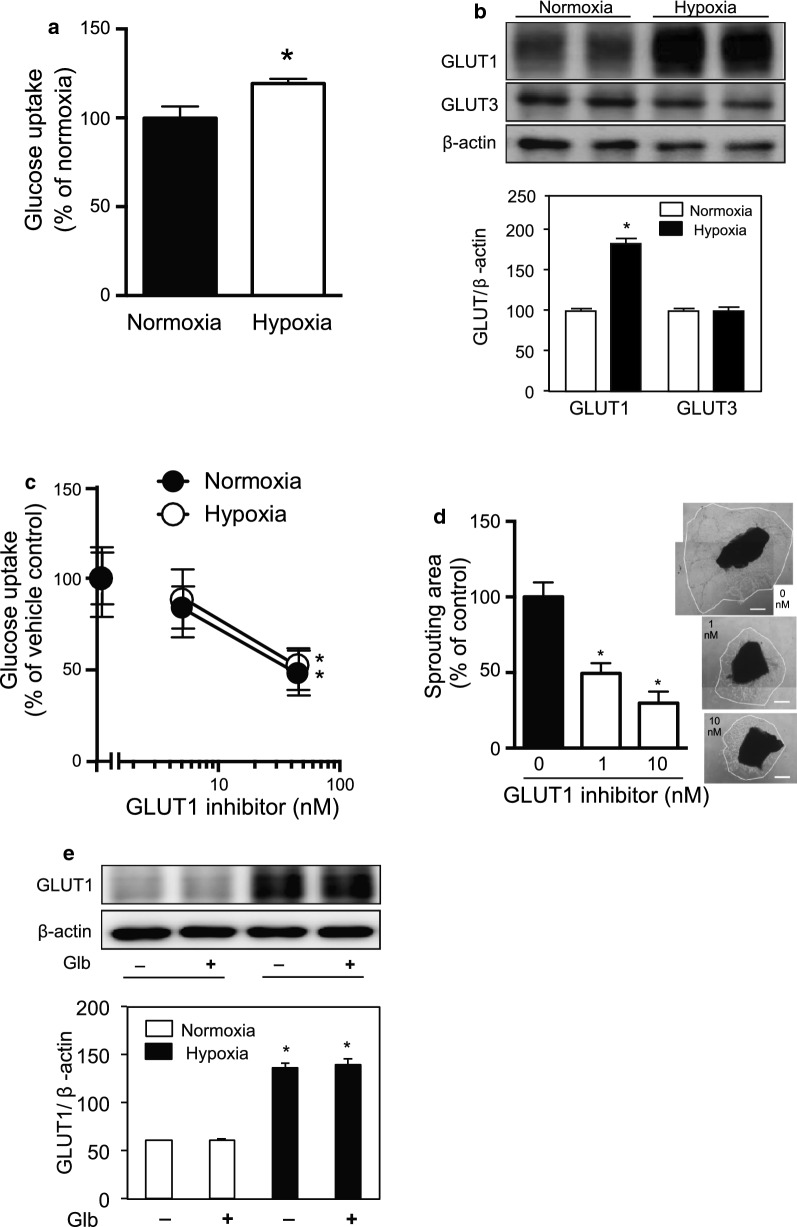
Glucose uptake and GLUT1 expression in HUVECs under normoxia and hypoxia. **a** Glucose uptake of HUVECs under normoxia and hypoxia. HUVECs were exposed to hypoxia, and glucose uptake was measured as described in the Materials and methods. Data are expressed as the mean ± SEM from 6 independent experiments. *P < 0.05 (Mann–Whitney test). **b** GLUT1 and GLUT3 expression in HUVECs under normoxia and hypoxia determined by immunoblotting. Data are expressed as the mean ± SEM of 12 samples from 3 independent experiments. *P < 0.01 vs. normoxia control of each condition (Mann–Whitney test). **c** Effect of the GLUT1 inhibitor BAY876 on glucose uptake in HUVECs. HUVECs were cultured under normoxia or hypoxia with BAY876 as described in the Materials and Methods. Data are expressed as a percentage of glucose uptake compared with vehicle control in each condition. Data are expressed as the mean ± SEM from 5 independent results. *P < 0.01 (one-way ANOVA). **d** Effect of GLUT1 inhibitor on sprouting of cells from choroid explants [16]. The explants were treated with vehicle or the GLUT1 inhibitor BAY876 for 4 days. Images were taken and the area of cell sprouting was quantified by ImageJ software. Data are the mean ± SEM from 12 explants in 2 time-independent experiments with similar results. *P < 0.01 (one-way ANOVA with Dunnett’s correction). **e** GLUT1 expression in HUVECs under normoxia and hypoxia determined by immunoblotting. Data are expressed as the mean ± SEM from 6 independent experiments. Glb, glibenclamide (10 μM). *P < 0.01 vs. normoxia control of each condition (Mann–Whitney test)

We first examined the influence of hypoxia on glucose uptake and GLUT expression in HUVECs. Glucose uptake was increased by 19% under hypoxia (1% O_2_, 12 h) (19.3 ± 2.6 vs. normoxia, mean ± SEM, *p* < 0.05) (Fig. 1a), which was associated with an increase in GLUT1 protein expression (Fig. 1b). At the mRNA level, an increase of GLUT1 and GLUT3 expression was detected; however, there was no increase in the expression of GLUT3 protein. The expression of GLUT2, GLUT4, and GLUT5 mRNA was not detected in HUVECs, as previously reported (Table 1) [18]. Glucose uptake was significantly reduced by the GLUT1 inhibitor BAY876 under normoxia and hypoxia (Fig. 1c). These data indicated the isoform-specific regulation of GLUTs and the importance of GLUT1 for glucose uptake in vascular endothelial cells under hypoxia.

**Table 1:** Expression of GLUT1, GLUT3, and KATP channel subunits in HUVECs under normoxia and hypoxia

Gene name	Normoxia	Hypoxia	*P* Value
	Mean ± SEM	Mean ± SEM	
GLUT1	0.483 ± 0.041	1.729 ± 0.149	< 0.01
GLUT3	0.708 ± 0.172	1.544 ± 0.148	< 0.05
Kir6.1	0.868 ± 0.080	1.044 ± 0.126	n.s.
Kir6.2	1.223 ± 0.823	0.844 ± 0.229	n.s.
SUR-1	0.997 ± 0.317	1.083 ± 0.122	n.s.
SUR-2A	1.117 ± 0.216	1.035 ± 0.130	n.s.
SUR-2B	0.899 ± 0.110	0.972 ± 0.039	n.s.

The mRNA levels of GLUTs and KATP channel subunits were analyzed by real-time PCR [11, 13]. Data are shown as relative values normalized with 18S RNA. Data are expressed as the mean ± SEM of 4 independent experiments. *P*-values were analyzed by an unpaired *t*-test. *n.s.* not significant

The role of GLUT1 was examined further using a sprouting assay of mouse choroid, which is an ex vivo model of angiogenesis (Fig. 1d) [16]. Cultured choroid fragments in the Matrigel (BD Biosciences) initiated the outgrowth of endothelial cells by VEGF [16]. The GLUT1 inhibitor BAY876 suppressed the outgrowth of cells from the explants, suggesting the importance of GLUT1 for the growth of endothelial cells and angiogenesis.

### Hypoxia induces GLUT1 translocation to the plasma membrane

**Fig. 2:**
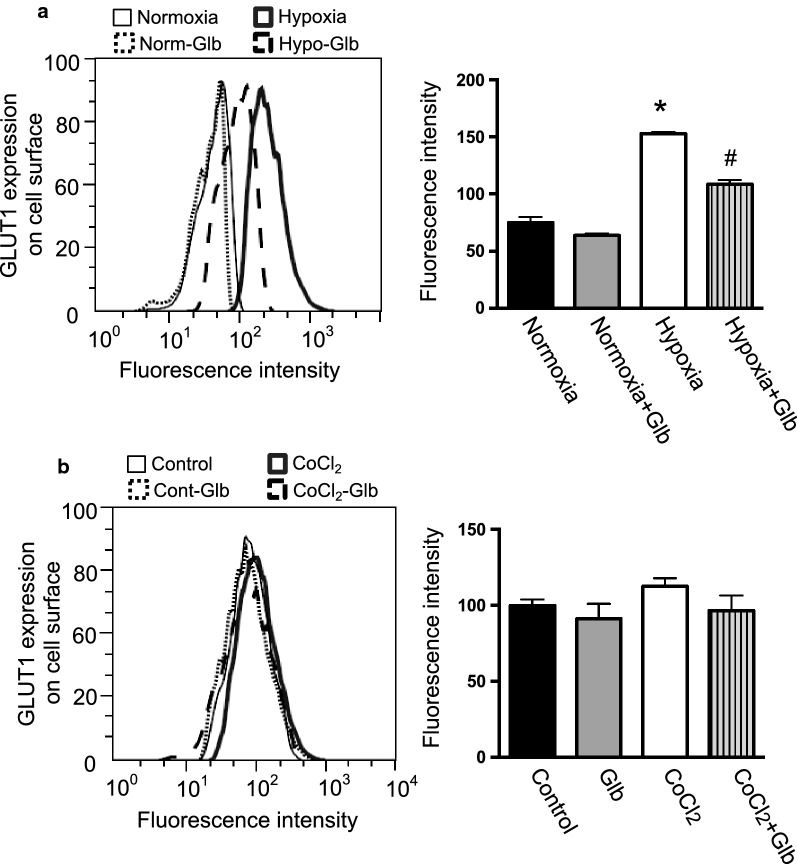
Effect of hypoxia or reagents on the cell surface expression of GLUT1. **a** HUVECs were cultured under normoxia or hypoxia as described in the Materials and Methods. In some experiments, the cells were treated with glibenclamide (Glb, 10 μM). The cells were labeled a human anti-GLUT1 Alexa Fluor 700-conjugated antibody to detect cell surface GLUT1. **b** Effect of CoCl_2_ and/or glibenclamide on the cell surface expression of GLUT1. HUVECs were treated with CoCl_2_ (100 µM) and/or glibenclamide (Glb, 10 μM). GLUT1 at the cell surface was determined as described in (**a**). (**a, b**) The histograms represent cell counts (Y-axis, linear scale) versus fluorescence intensity (X-axis, log scale). Fluorescence mean intensity obtained from the histograms was quantified and shown in bar graphs (right panels). Data are the mean ± SEM from 4 independent experiments. *P < 0.01 versus normoxia control (Mann–Whitney test). ^#^*P* < 0.01 versus hypoxia without glibenclamide treatment (Mann–Whitney test)

**Fig. 3:**
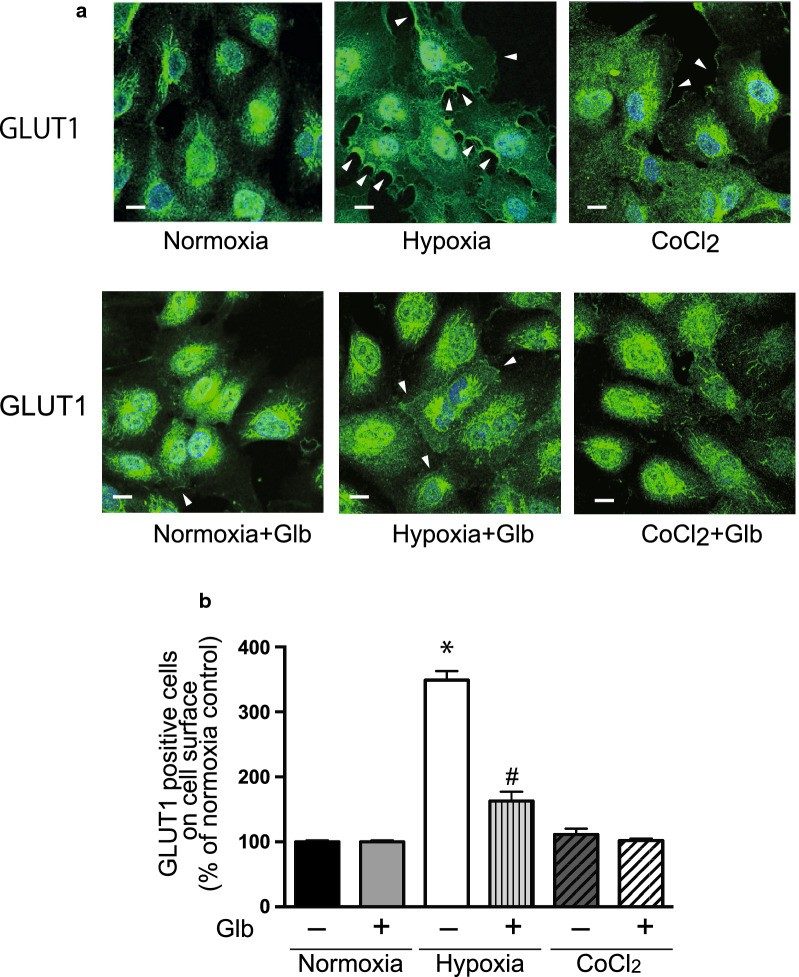
GLUT1 expression on the cell surface. **a** HUVECs were cultured under normoxia or hypoxia as described in the Materials and Methods. In some experiments, the cells were treated with glibenclamide (Glb, 10 μM). **a** The cellular distribution of GLUT1 was determined by immunofluorescence staining. HUVECs were fixed with 4% paraformaldehyde and stained with an anti-GLUT1 antibody (green). Images were obtained with a confocal fluorescence microscope as described in the Materials and Methods. Representative images are shown from 4 independent experiments with similar results. Scale bars, 25 μm. **b** The number of cells in which GLUT1 was observed at the cell membrane was counted in approximately 100 cells from 4 or 5 independent experiments. Data are the mean ± SEM from 4 independent experiments. *P < 0.01 versus normoxia control (unpaired *t*-test). ^#^*P* < 0.01 versus hypoxia without glibenclamide treatment (unpaired *t*-test)

**Fig. 4:**
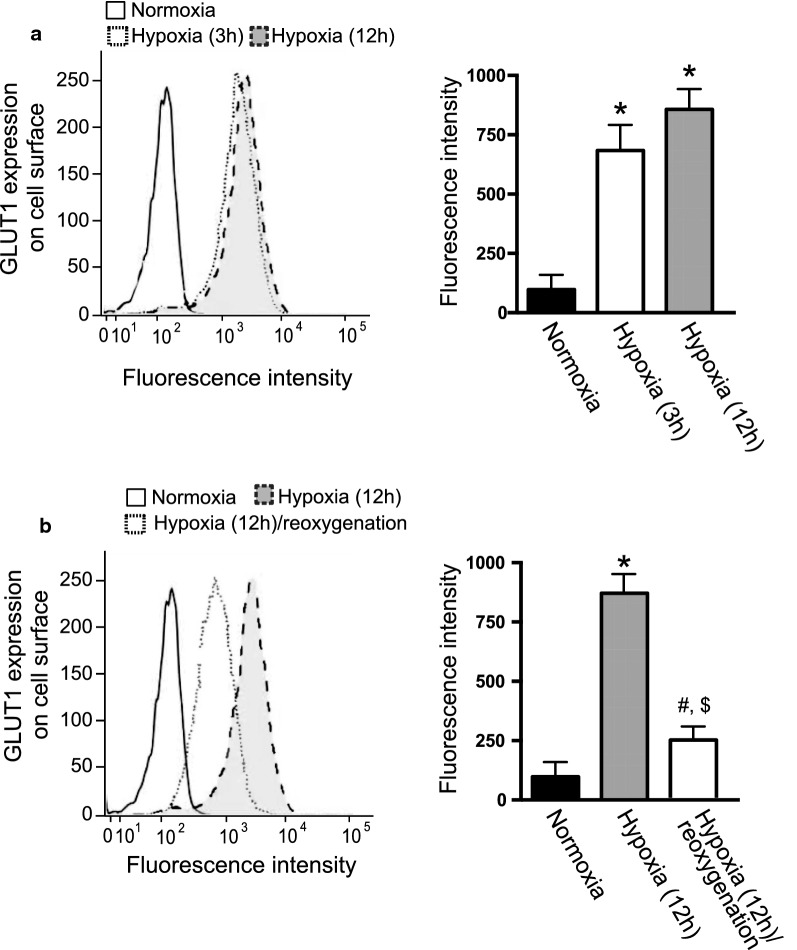
Effect of hypoxia and reoxygenation on the cell-surface expression of GLUT1. **a** HUVECs were cultured under normoxia or 3 h or 12 h hypoxia, as described in the Materials and Methods. The cells were labeled with a human anti-GLUT1 Alexa Fluor^®^ 488-conjugated antibody to detect cell-surface GLUT1. **b** Effect of re-oxygenation on the cell-surface expression of GLUT1. HUVECs were cultured under 12 h hypoxia or 12 h normoxia following 12 h hypoxia. GLUT1 at the cell surface was determined as described in **a**. (**a, b**) The histograms represent cell counts (Y-axis, linear scale) versus fluorescence intensity (X-axis, log scale). Fluorescence mean intensity obtained from the histograms was quantified and shown in bar graphs (right panels). Data are the mean ± SEM from 4 independent experiments. *P < 0.01, ^#^*P* < 0.05 vs. normoxia control (Mann–Whitney test). ^$^*P* < 0.01 vs. hypoxia (12 h) (Mann–Whitney test)

In addition to the cellular expression of GLUT1, its cell surface localization is another critical factor influencing glucose uptake [2]. The cell surface localization of GLUT1 was analyzed by FACS using a fluorescent dye-conjugated anti-GLUT1 antibody without permeabilization of the cells. The GLUT1 signal on the cell surface was significantly increased by exposure of HUVECs to hypoxia (Fig. 2a). The cell surface localization of GLUT1 was confirmed by fluorescent immunostaining in HUVECs (Fig. 3). The GLUT1 signal was increased at the cell surface under hypoxia (Fig. 3a, b). These data suggest that hypoxia triggers the translocation of GLUT1 to the cell surface. Interestingly, GLUT1 was increased at the cell surface, even after 3-h exposure of the cells to hypoxia (Fig. 4a), and was markedly decreased by reoxygenation (Fig. 4b), suggesting the dynamic regulation of cell-surface GLUT1 in response to the oxygen levels of cells.

### Decrease of ATP triggers GLUT1 translocation to the plasma membrane

**Fig. 5:**
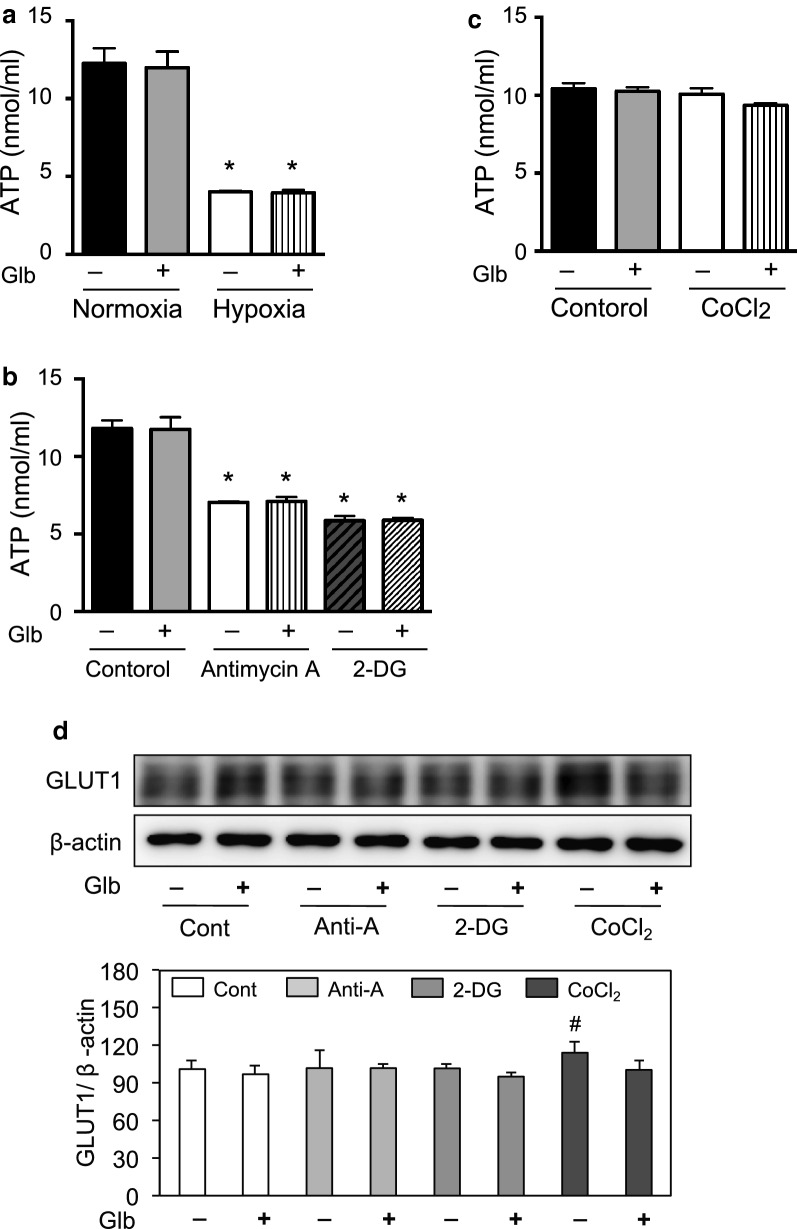
ATP concentration and GLUT1 expression in HUVECs. **a** Intracellular ATP production was measured following culture of the cells under normoxia or hypoxia, with or without glibenclamide (Glb, 10 μM), as described in the Materials and Methods. Data are expressed as the mean ± SEM from 4 independent experiments. *P < 0.01 vs. normoxic control without glibenclamide treatment (Mann–Whitney test). **b** HUVECs in 96-well plates were treated with antimycin-A (Anti-A, 10 μM) or 2-deoxyglucose (2-DG, 1 mM) for 30 min at normoxia. In some groups, the cells were also treated with glibenclamide (Glb, 10 μM). ATP was measured as described in the Materials and Methods. Data are from experiments performed in duplicate. *P < 0.01 compared with control without glibenclamide treatment (Mann–Whitney test). **c** HUVECs were treated with CoCl_2_ (100 μM) with or without glibenclamide (Glb, 10 μM). Intracellular ATP concentration was analyzed as described in the “Materials and methods”. Data are expressed as the mean ± SEM from 4 independent experiments. No statistical significance was detected by the Mann–Whitney test. **d** GLUT1 expression determined by immunoblotting following treatment with antimycin-A (Anti-A, 10 μM), 2-deoxyglucose (2-DG, 1 mM), CoCl_2_ (100 µM), and/or glibenclamide (Glb, 10 μM). Data are expressed as the mean ± SEM from 4 independent experiments. #*P* < 0.05 versus vehicle control without glibenclamide treatment (Mann–Whitney test)

**Fig. 6:**
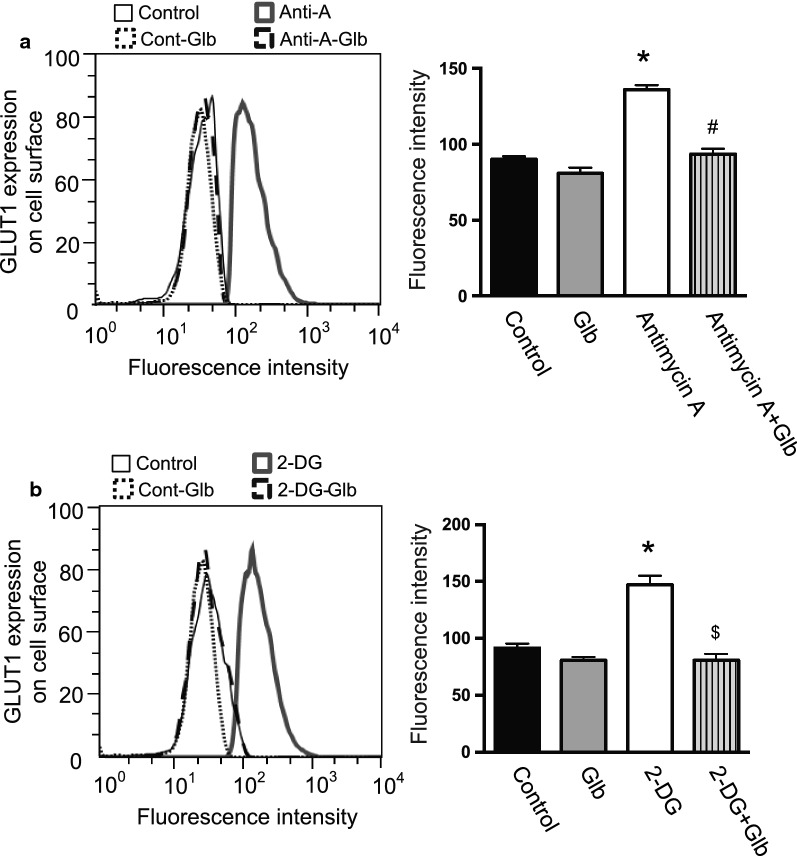
Effects of reagents decreasing cellular ATP levels on the cell surface expression of GLUT1. HUVECs were treated with antimycin-A (Anti-A, 10 μM) (**a**) or 2-deoxyglucose (2-DG, 1 mM) (**b**) for 30 min at normoxia. In some groups, the cells were also treated with glibenclamide (Glb, 10 μM). The cells were labeled with a human anti-GLUT1 Alexa Fluor 700-conjugated antibody to detect GLUT1 on the cell surface. (**a, b**) The histograms represent cell counts (Y-axis, linear scale) versus fluorescence intensity (X-axis, log scale). Fluorescence mean intensity obtained from the histograms was quantified and shown in bar graphs (right panels). Data are the mean ± SEM from 4 independent experiments. *P < 0.01 versus normoxia control (Mann–Whitney test). ^#^*P* < 0.01 versus antimycin-A (Mann–Whitney test). ^$^*P* < 0.01 versus 2-deoxyglucose (Mann–Whitney test)

**Fig. 7:**
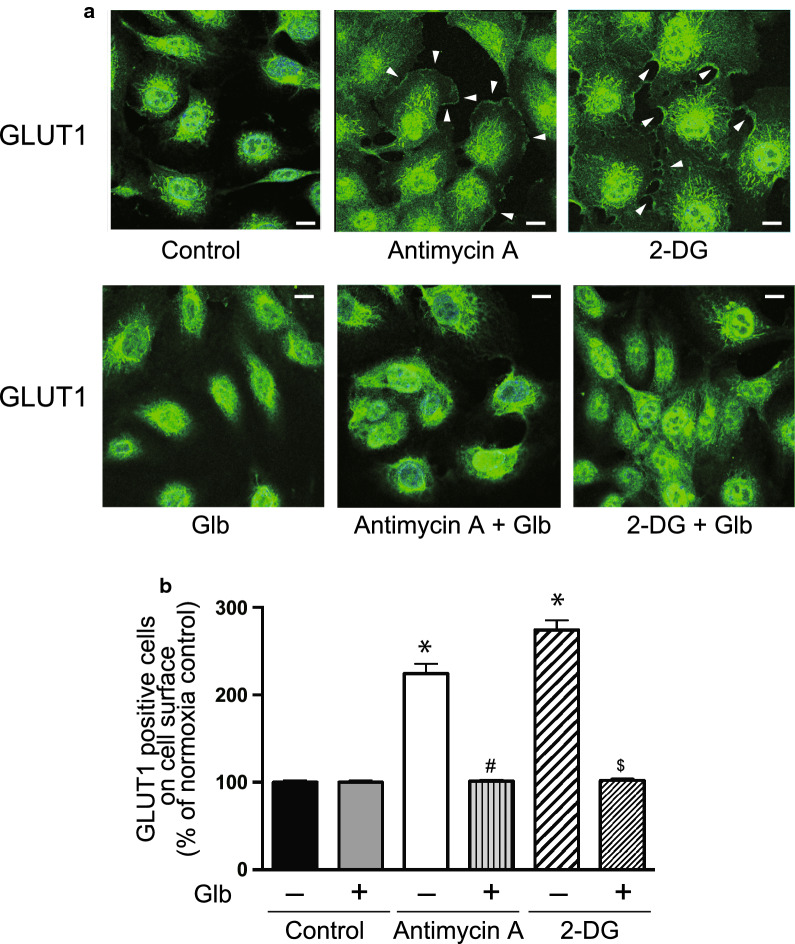
Effects of reagents decreasing cellular ATP levels on the subcellular localization of GLUT1. **a** HUVECs were treated with antimycin-A (Anti-A, 10 μM) or 2-deoxyglucose (2-DG, 1 mM) with or without glibenclamide (Glb, 10 μM) under normoxia. The cellular distribution of GLUT1 was determined by immunofluorescence staining as described in the Materials and Methods. HUVECs were stained with an anti-GLUT1 antibody (green). Images were obtained with a confocal fluorescence microscope as described in the “Materials and methods”. Representative images are shown from 4 independent experiments with similar results. Scale bars, 25 μm. **b** The number of cells in which GLUT1 was observed at the cell membrane was counted in approximately 100 cells from 4 or 5 independent experiments. Data are the mean ± SEM from 4 independent experiments. *P < 0.01 versus normoxia control without glibenclamide treatment (unpaired *t*-test). ^#^*P* < 0.01 versus antimycin-A without glibenclamide treatment (unpaired *t*-test). ^$^*P* < 0.01 versus 2-deoxyglucose without glibenclamide treatment (unpaired *t*-test)

Cellular ATP was decreased by exposing the cells to hypoxia as reported previously (Fig. 5a) [11], which may be associated with GLUT1 translocation. The cell surface expression of GLUT1 was determined in the presence of antimycin-A or 2-DG, because they decrease cellular ATP even under normoxia (Fig. 5b) [11]. Interestingly, FACS analysis indicated that antimycin-A and 2-DG induced GLUT1 translocation to the plasma membrane (Fig. 6). This was also confirmed by immunofluorescence staining of GLUT1 (Fig. 7), suggesting that a decrease of ATP was key for GLUT1 translocation.

### KATP channels are involved in hypoxia-induced GLUT1 translocation

To identify which molecule triggers the translocation of GLUT1 associated with a reduction of ATP, we examined KATP channels, which open following a decrease of intracellular ATP. HUVECs expressed KATP channel subunits, but their levels were not changed significantly during hypoxia (Table 1). Next, we analyzed the effect of glibenclamide, which blocks KATP channels, on GLUT1 translocation by FACS analysis and immunofluorescence staining (Figs. 2a and 3). Interestingly, glibenclamide inhibited the hypoxia-induced translocation of GLUT1 to the cell surface in both analyses (Figs. 2a and 3). Furthermore, glibenclamide also inhibited the translocation of GLUT1 triggered by a decrease of ATP following treatment with antimycin-A and 2-DG under normoxia (Figs. 6 and 7). Treatment with glibenclamide reportedly reduces the expression of GLUT1 in human chondrocytes [13]; however, in HUVECs, it did not influence GLUT1 expression or intracellular ATP concentration (Figs. 1e and 5a–d). These data indicated that KATP channels are involved in the hypoxia-induced translocation of GLUT1 by sensing a decrease of ATP levels.

### Mitochondrial KATP channels are not involved in GLUT1 translocation

**Fig. 8:**
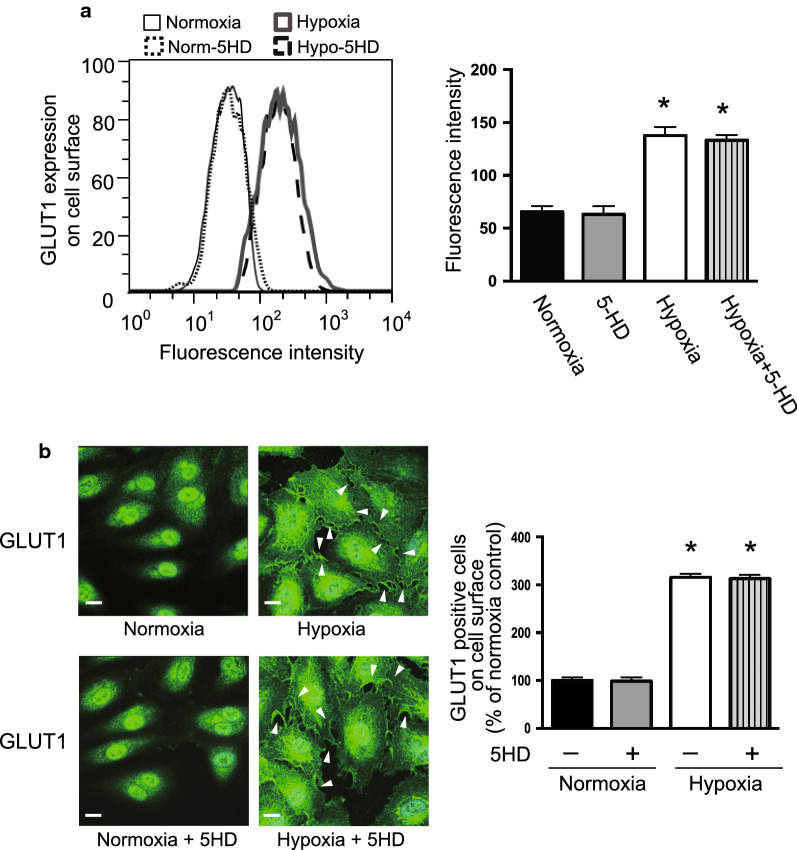
Effects of the mitochondrial KATP channel inhibitor 5-HD on the cell surface expression of GLUT1. HUVECs were cultured under normoxia or hypoxia with or without 5-HD (100 μM). **a** Cell surface expression of GLUT1 determined by flow cytometric analysis. The cells were labeled with a human anti-GLUT1 Alexa Fluor 700-conjugated antibody to detect cell surface GLUT1. Left panel: The histograms represent cell counts (Y-axis, linear scale) versus fluorescence intensity (X-axis, log scale). Right panel: Fluorescence mean intensity obtained from the histograms was quantified and shown in bar graphs (right panels). Data are the mean ± SEM from 4 independent experiments. *P < 0.01 versus normoxia control (Mann–Whitney test). **b** The cellular distribution of GLUT1 was determined by immunofluorescence staining as described in the Materials and Methods. HUVECs were stained with an anti-GLUT1 antibody (green). Left panel: Images were obtained with a confocal fluorescence microscope as described in the “Materials and methods”. Representative images are shown from 4 independent experiments with similar results. Scale bars, 25 μm. Right panel: The number of cells in which GLUT1 was observed at the cell membrane was counted in approximately 100 cells from 4 independent experiments. Data are the mean ± SEM from 4 independent experiments. *P < 0.01 versus normoxia control (Mann–Whitney test)

Glibenclamide blocks KATP channels in the plasma membrane and other organelles including mitochondria [19, 20]. Therefore, we examined the role of mitochondrial KATP channels on the hypoxia-induced translocation of GLUT1. Interestingly, the mitochondrial KATP channel blocker 5-HD did not influence hypoxia-induced GLUT1 translocation, as determined by FACS analysis and immunofluorescence staining (Fig. 8). These data indicated that KATP channels in the plasma membrane or other organelles, but not in mitochondria, are involved in the hypoxia-induced translocation of GLUT1.

### HIF-1α does not induce GLUT1 translocation

A number of genes that are induced under hypoxia may also have a role in GLUT1 translocation. Hypoxia inducible factor-1 (HIF-1) is an important regulator of the metabolic adaptation of cells to hypoxia. CoCl_2_ stabilizes HIF-1α and mimics the hypoxic environment in cells [21]. CoCl_2_ increased GLUT1 expression (Fig. 5d) [11]; however, it did not influence intracellular ATP concentration (Fig. 5c) or GLUT1 translocation, as determined by FACS analysis and immunofluorescence staining (Figs. 2b and 3). These data indicated that the increased cellular expression of HIF-1α did not induce a change in the level of GLUT1 in the plasma membrane, but another signal was required to trigger GLUT1 translocation to the plasma membrane.

## Discussion

In this study, we demonstrated that the exposure of endothelial cells to hypoxia induced the translocation of GLUT1 to the cell surface. GLUT1 translocation was associated with a decrease of intracellular ATP, but not an increase of HIF-1. Further analysis suggested that KATP channels were involved in GLUT1 translocation. The ATP-triggered translocation of GLUT1 to the plasma membrane may be a part of a feedback mechanism between metabolic status and glucose uptake, which could contribute to glucose uptake during hypoxia and the angiogenic events of endothelial cells.

Glucose uptake and glycolysis are critical processes for energy production in endothelial cells. Glucose uptake was mediated by GLUT1 and GLUT3 in HUVECs (Table 1) [18]. GLUT3 is predominantly expressed in neurons and may play a role in angiogenesis in glioblastoma [22]. GLUT1 is the most important glucose transporter for a variety of cells [3, 4, 23]. In HUVECs, the GLUT1 inhibitor BAY876 significantly reduced glucose uptake and suppressed the outgrowth of endothelial cells from explants (Fig. 1), suggesting the importance of GLUT1 for angiogenic events in endothelial cells. Under hypoxia, GLUT1 expression was increased to facilitate glucose uptake. In addition to the levels of GLUTs, their cell surface localization is another determinant of glucose uptake [2]. In a series of experiments, the increase of cellular GLUT1 expression induced by HIF-1 was not concomitant with an increase of GLUT1 in the plasma membrane (Figs. 2b, 3, and 5d), suggesting that another signal was required to trigger GLUT1 translocation. However, GLUT1 trafficking in endothelial cells has not been characterized in detail.

Hypoxia significantly increased the cell surface localization of GLUT1 in HUVECs. The translocation of GLUT4 has been characterized in detail, in which its translocation is induced by the activation of insulin receptors and the subsequent activation of the phosphatidylinositol 3-kinase (PI3K) and AKT pathway [2]. Several factors are reported to induce the translocation of GLUT1 to the plasma membrane, including interleukin 3 (IL-3) [24], nitric oxide [25], low-density lipoprotein (LDL) [26], insulin [27], dehydroepiandrosterone (DEHA) [28], ischemia [27], and the combination of hypoxia and high glucose concentration [29]. PI3K inhibition [28] and AKT knockdown [30] block the translocation of GLUT1 induced by insulin [28, 30] and DEHA [28], as observed for GLUT4. Similarly, PI3K inhibition also attenuates the translocation of GLUT1 induced by IL-3 [24], LDL, and the combination of hypoxia with high glucose concentration [29]. However, PI3K inhibition fails to block the translocation of GLUT1 induced by ischemia, suggesting the existence of another pathway regulating GLUT1 trafficking [27]. In this study, hypoxia clearly induced GLUT1 translocation in HUVECs, which was associated with a decrease of ATP concentration. A decrease of intracellular ATP was also related to the translocation of GLUT1 to the plasma membrane in a malaria-infected hepatocyte cell line [31], suggesting the existence of a mechanism triggered by an ATP-sensing molecule leading to GLUT1 translocation.

KATP channels are potential ATP-sensing molecules [32]. KATP channels consist of a large macromolecular complex in which four inwardly rectifying potassium channel (Kir6.x) subunits form a central pore surrounded by four regulatory sulphonyl urea receptor (SUR) subunits [33, 34]. Changes in intracellular ATP concentrations regulate KATP channel activity; higher ATP concentrations in cells inhibit or close KATP channels by binding to Kir6.x, while Mg-nucleotide binding/hydrolysis at the nucleotide-binding domains of SUR stimulates channel opening [35]. The balance between these stimulatory and inhibitory effects determines the level of channel activity. HUVECs expressed the necessary components to assemble functional KATP channels, and their levels were not changed under hypoxia compared with normoxia (Table 1). Glibenclamide, which binds to SUR subunits and inhibits KATP channel activity in the plasma membrane and other organelles including mitochondria, inhibited the hypoxia-induced translocation of GLUT1. However, 5-HD, a mitochondrial KATP channel inhibitor, had no effect on GLUT1 translocation during hypoxia. These data suggested that KATP channels in the plasma membrane or other organelles rather than mitochondria are involved in the hypoxia-induced translocation of GLUT1. The opening of KATP channels in the plasma membrane, stimulated by low levels of intracellular ATP, typically decreases the membrane potential of endothelial cells. Currently, the mechanism by which hyperpolarization induces the translocation of molecules is not well-defined and thus further studies are required. In addition to KATP channels in the plasma membrane, the presence of KATP channels in the endoplasmic reticulum and nuclear membrane has also been reported [19, 20, 36]. KATP channels in such organelles may be involved in the translocation of GLUT1 under hypoxia.

## Conclusion

In conclusion, we have demonstrated the isoform-specific expression of GLUTs and the translocation of GLUT1 in vascular endothelial cells under hypoxia. GLUT1 translocation was induced by a decrease of intracellular ATP and was mediated by KATP channels. Glucose uptake and subsequent ATP production are important for angiogenic events in endothelial cells. Our data indicate the existence of functional feedback between metabolic status and GLUT1 translocation, which will contribute to a more precise understanding of the induction of angiogenesis by hypoxia and tumors.

## Data Availability

All relevant data are within the paper.
